# Evaluation of dihydrotestosterone and dihydroprogesterone levels and gene expression of genes involved in neurosteroidogenesis in the SH-SY5Y Alzheimer disease cell model

**DOI:** 10.3389/fnins.2023.1163806

**Published:** 2023-05-23

**Authors:** Saeed Radagdam, Fatemeh Khaki-Khatibi, Reza Rahbarghazi, Behrouz Shademan, Seyed Manouchehr Nourazarian, Masoud Nikanfar, Alireza Nourazarian

**Affiliations:** ^1^Department of Biochemistry and Clinical Laboratories, Faculty of Medicine, Tabriz University of Medical Sciences, Tabriz, Iran; ^2^Stem Cell Research Center, Tabriz University of Medical Sciences, Tabriz, Iran; ^3^Department of Applied Cell Sciences, Faculty of Advanced Medical Sciences, Tabriz University of Medical Sciences, Tabriz, Iran; ^4^Imam Reza Medical Research and Training Hospital, Tabriz University of Medical Sciences, Tabriz, Iran; ^5^Department of Neurology, Faculty of Medicine, Tabriz University of Medical Sciences, Tabriz, Iran; ^6^Department of Basic Medical Sciences, Khoy University of Medical Sciences, Khoy, Iran

**Keywords:** Alzheimer’s disease, lipopolysaccharide, dihydrotestosterone, dihydroprogesterone, human neurogenesis

## Abstract

**Introduction:**

Alzheimer’s disease (AD) is the most common form of dementia worldwide. This study investigated the effects of lipopolysaccharide on neurosteroidogenesis and its relationship to growth and differentiation using SH-SY5Y cells.

**Methods:**

In this study, we used the MTT assay to assess the impact of LPS on SH-SY5Y cell viability. We also evaluated apoptotic effects using FITC Annexin V staining to detect phosphatidylserine in the cell membrane. To identify gene expression related to human neurogenesis, we utilized the RT^2^ Profiler TM PCR array human neurogenesis PAHS-404Z.

**Results:**

Our study found that LPS had an IC50 level of 0.25 μg/mL on the SH-SY5Y cell line after 48 h. We observed Aβ deposition in SH-SY5Y cells treated with LPS, and a decrease in DHT and DHP levels in the cells. Our analysis showed that the total rate of apoptosis varied with LPS dilution: 4.6% at 0.1 μg/mL, 10.5% at 10 μg/mL, and 44.1% at 50 μg/mL. We also observed an increase in the expression of several genes involved in human neurogenesis, including ASCL1, BCL2, BDNF, CDK5R1, CDK5RAP2, CREB1, DRD2, HES1, HEYL, NOTCH1, STAT3, and TGFB1, after treatment with LPS at 10 μg/mL and 50 μg/mL. LPS at 50 μg/mL increased the expression of FLNA and NEUROG2, as well as the other genes mentioned.

**Conclusion:**

Our study showed that LPS treatment altered the expression of human neurogenesis genes and decreased DHT and DHP levels in SH-SY5Y cells. These findings suggest that targeting LPS, DHT, and DHP could be potential therapeutic strategies to treat AD or improve its symptoms.

## 1. Introduction

The expression of Aβ, tau, synaptic factors, and other neuron-specific proteins in SH-SY5Y cells makes them a suitable model to study the mechanism of neuron phenotype degeneration, including AD (Aitken et al., 2016). AD is a neurological disorder and the most common cause of dementia in older adults (Hoseinlar et al., 2023; Shademan et al., 2023). Although more common in women, it can occur in both men and women. Statistically, there are estimated 5.2 million people in the United States with the disease, including 3.3 million women and 1.9 million men (Aslanpour et al., 2020a). Several studies have been conducted to treat and prevent AD, but a definitive treatment has not yet been approved. Drug treatment and medical costs are three times higher for people with Alzheimer’s disease than for others. Factors such as immobility, high blood sugar, cholesterol levels, and genetic history can play a role in the development of AD (Aslanpour et al., 2020b). The neuropathology of Alzheimer’s disease is mainly affected by extracellular accumulation of amyloid beta (Aβ), accumulation of tau in neurons, glial activation, and loss of neurons and synapses (Hoseinlar et al., 2023). Neuropathological changes are associated with glial activity that causes nerve damage, loss of synapses, and neuronal death (Lawrence et al., 2023). Although the mechanisms underlying AD pathogenesis are not fully understood, amyloid plaques are thought to be involved in disease progression. The primary precursors for forming Aβ-plaques are the amyloid beta protein and neurofibrillary tau tangles in the brain. The primary amyloid precursor performs many functions, from neurotransmission to gene transcription (Beydoun et al., 2021).

Pregnant mice exposed to repeated systemic exposure to LPS (lipopolysaccharide) cause Alzheimer’s disease-related features, including behavioral and neuropathological changes, in their offspring (Wang et al., 2020). Despite the limited and temporary induction of neuronal damage, a single systemic challenge of LPS (lipopolysaccharide) leads to increased deposition of Aβ1-42 and tau levels in the brains of wild-type rodents (Wang et al., 2018). Moreover, repeated systemic injections of LPS (lipopolysaccharide) can lead to prolonged elevation of Aβ levels and cognitive deficits (Xie et al., 2022).

Sex hormones can influence growth, synaptogenesis, dendritic branching, and myelination (Calan et al., 2016), and other important mechanisms of neuroplasticity (Calan et al., 2016). Sex hormones are released into various tissues and the brain via the bloodstream. In addition to the sex glands, they are also produced in the brain and can improve brain function (Carroll et al., 2010). Sex hormones can regulate neuronal survival in different areas of the central nervous system (CNS) and promote repair of neuronal damage (Castro et al., 2011). Sex hormones can affect different areas of the CNS, including the brain, spinal cord, and peripheral nerves, due to the presence of estrogen and progesterone receptors in these sites. The increased risk of AD is significantly influenced by sexual steroid hormones in women. Decreased levels of progesterone are associated with an increased risk of AD. In men, the risk of developing AD is also affected by the male sex steroid hormone testosterone. The rate of testosterone production gradually decreases with age, leading to an increased risk of developing AD (Radaghdam et al., 2021). There is still debate about whether sex hormones should be used as a treatment or prevention strategy for diseases associated with neurological damage, including AD. However, progesterone and testosterone can reduce AD in several ways, including the MAPK/ERK and CREB pathways (Chouchane and Costa, 2019).

LPS exposure can lead to various chronic diseases and induce mitochondrial apoptosis in neurodegenerative conditions. Sirtuin 1 plays a key role in the function of testosterone and estrogen receptors, along with androgens and estrogens. The concentration of LPS may affect the expression of the Sirtuin 1 gene, which in turn may be related to the production of dihydrotestosterone (DHT) and dihydroprogesterone (DHP), as well as neurogenesis. Therefore, the effect of LPS concentration on gene expression may be associated with DHT and DHP (Ian, 2017; Martins, 2018; Rasha et al., 2020; Tsuchiya et al., 2020). Identification of the genes involved in these mechanisms can aid in the development of treatments and management strategies for AD.

Examination of changes in the expression of genes related to human neuroblastoma neurogenesis in SH-SY5Y cells under the influence of LPS may offer new information on the submechanisms involved. This study aimed to investigate the effect of lipopolysaccharide (LPS) on neurosteroidogenesis and its relationship with growth and differentiation, as well as the expression patterns of genes involved in neurogenesis in the human neuroblastoma cell line, SH-SY5Y.

## 2. Materials and methods

### 2.1. Cell line, culture conditions, and chemicals

The SH-SY5Y cell line was obtained from the cell bank of the Pasteur Institute (Tehran, Iran). The cells were cultured in Dulbecco’s modified Eagle Medium/Nutrient Mixture F-12 medium (DMEM-F12, Gibco) supplemented with 10% fetal bovine serum (FBS, Gibco), 1% penicillin (100 units/mL, Biosera), streptomycin (100 μg/mL, Biosera), and 250 μmol/L cholesterol. The cells were incubated at 37°C with 95% humidity and 5% CO_2_. After 48 h, cells were harvested at 70–80% confluence for further analysis. To prepare a concentration of 250 μmol/L cholesterol, we dissolved 9.6 mg of cholesterol in 1 cc of ethanol and diluted at 1:100 with RPMI medium. The cells were then treated with different concentrations of LPS, including 0.1, 10, and 50 μg/mL (as a control group) for 24, 48, and 72 h, respectively. Based on the results of the MTT assay, we selected LPS 10 μg/mL for subsequent analyses.

### 2.2. Cell viability assay

We used the MTT assay (Gibco, United States) to assess the effect of LPS (Chen and Song, 2020) on cell viability in SH-SY5Y cells. SH-SY5Y cells were seeded in 96-well plates at a density of 1 × 104 cells/well and incubated for 24 h under standard conditions. The cells were then treated with different concentrations of LPS (0.1, 10, and 50 μg/mL) for 24, 48, and 72 h. The medium was replaced with 100 μL of MTT solution (5 mg/mL in PBS) and incubated for 4 h under standard conditions. Subsequently, 50 μL of dimethyl sulfoxide (DMSO; Merck) was added to each well and the plates were incubated for 30 min at 37°C. LPS IC50 values for LPS in the SH-SY5Y cell line were determined after 48 h, and subsequent experiments were performed using this concentration. The optical density (OD) of the wells was measured at 570 nm using a microplate reader (BioTek, United States), and cell viability was evaluated based on the results.

### 2.3. Deposition of amyloid beta

The control group (SH-SY5Y cells cultured in a medium without cholesterol and LPS) and the case group (treated with LPS) were used to examine amyloid beta deposits with Congo staining. Amyloid detection was performed according to the instructions of the Vitro View Congo Red Amyloid Stain Kit (Biotech No. VB-3011, SKU). The CKX53 inverted microscope (Olympus) with a built-*in camera* port was used to detect amyloid deposits. When amyloid deposits were present in the environment, they became visible in red, while the nuclei appeared blue.

### 2.4. Measurement of dihydrotestosterone and dihydroprogesterone in SH-SY5Y cell line

DHT (Cat. No: MBS762135, MyBioSource, United States) and DHP (Cat No: DElA1592, Creative Diagnostics, United States), were quantified by the competitive ELISA detection method according to the manufacturer’s instructions. For this purpose, 1 × 10^4^ cells/well were plated in 96-well plates and treated with different concentrations of LPS (0.1, 10, and 50 μg/mL). Cell culture supernatant was centrifuged at 1000 × g and 2–8°C for 20 min to remove insoluble contaminants and cell debris. We diluted the buffer with a protease inhibitor (1:2) to prevent protein degradation. The OD was measured using a microplate reader (BioTek, United States) at a wavelength of 450 nm.

### 2.5. Evaluation of apoptosis

The apoptotic effects of the drugs on the cell lines based on phosphatidylserine in the cell membrane were evaluated using the FITC Annexin V apoptosis detection method, following the protocol. For this purpose, 1 × 10^4^ cells/well were counted on the BD ACCURI C6 flow cytometer (BD Biosciences Pharmingen). The data obtained from flow cytometry were analyzed using FlowJo software version 7.6.1. The apoptotic effects of the drugs were examined 48 h after they were added to cells at the indicated doses. The groups that were not treated with the drugs served as controls.

### 2.6. Determination of gene expression changes

SH-SY5Y cells were seeded in a 6-well plate (1 × 10^4^ cells/well) and incubated for 24 h under standard conditions. The expression changes induced by the IC50 doses of LPS in the SH-SY5Y cell line were determined using real-time polymerase chain reaction for 48 h. Total RNA was obtained from SH-SY5Y cells using the RNeasy kit (Cat. No.: FABRK001, Taiwan). Complementary DNA synthesis was performed using an RT2 first-strand kit (Cat. No.: YT4500, Austria). Changes in the expression of 96 genes associated with neurogenesis were examined using the RT2 Profiler TM Human Neurogenesis PCR Array (Qiagen, Cat. No.: PAHS-404Z) and the Light Cycler 480 instrument II (Roche). Data were analyzed using the comparative 2^−ΔΔCT^ method (Light Cycler 480 Quantification Software) using the changes made. Differences of more than ±2-fold change in expression were considered cut-off values with *p*-values <0.05.

### 2.7. Statistical analysis

Statistical analyses were performed using SPSS version 16. Differences between the treated groups and control groups were analyzed with one-way ANOVA, followed by Tukey’s *post hoc* analyses. All variables are expressed as mean ± SD. Statistical significance was established at *p* < 0.05.

## 3. Results

### 3.1. Evaluation of the effect of LPS on cell viability in SH-SY5Y cell line

**Figure 1:**
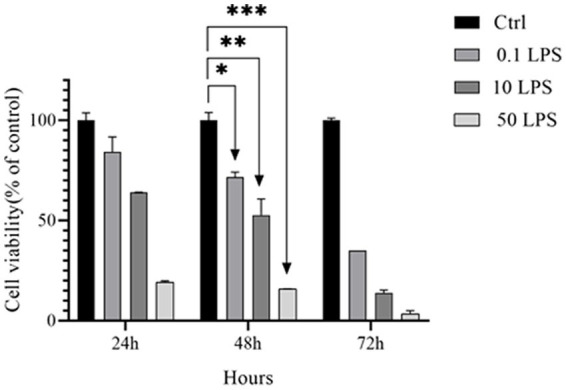
IC50 of LPS in SH-SY5Y cells. The MTT assay was used to determine the viability of SH-SY5Y cells treated with different concentrations of LPS for 24, 48, and 72 h (*p < 0.05, **p < 0.01, and ***p < 0.001). The data is presented as the mean ± SD of triplicate experiments.

To determine the value of LPS IC50, LPS dilutions of 0.1, 10, and 50 μg/mL were tested by the MTT assay at 24, 48, and 72 h in the SH-SY5Y cell line. The IC50 values of LPS for the SH-SY5Y cell line were 0.25 μg/mL at 48 h. Our results showed that cell death increased with increasing LPS dose (Figure 1).

### 3.2. Investigating the effect of LPS on the deposition of beta-amyloid

**Figure 2:**
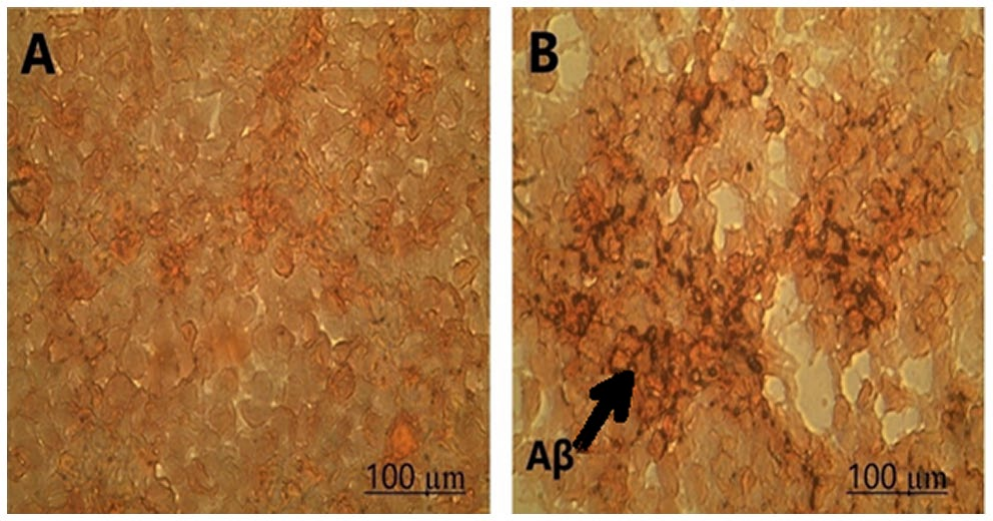
Amyloid-beta deposition was observed in **(A)** control and **(B)** SH-SY5Y cells treated with LPS using Congo staining at 100X magnification.

Congo red staining was performed to investigate Aβ deposition. The results indicated that LPS increased Aβ toxicity in SH-SY5Y cells, as amyloid beta deposition was observed in the cells treated with LPS (Figure 2).

### 3.3. Evaluation of changes in DHT and DHP concentrations induced by LPS in SH-SY5Y cell line

**Figure 3:**
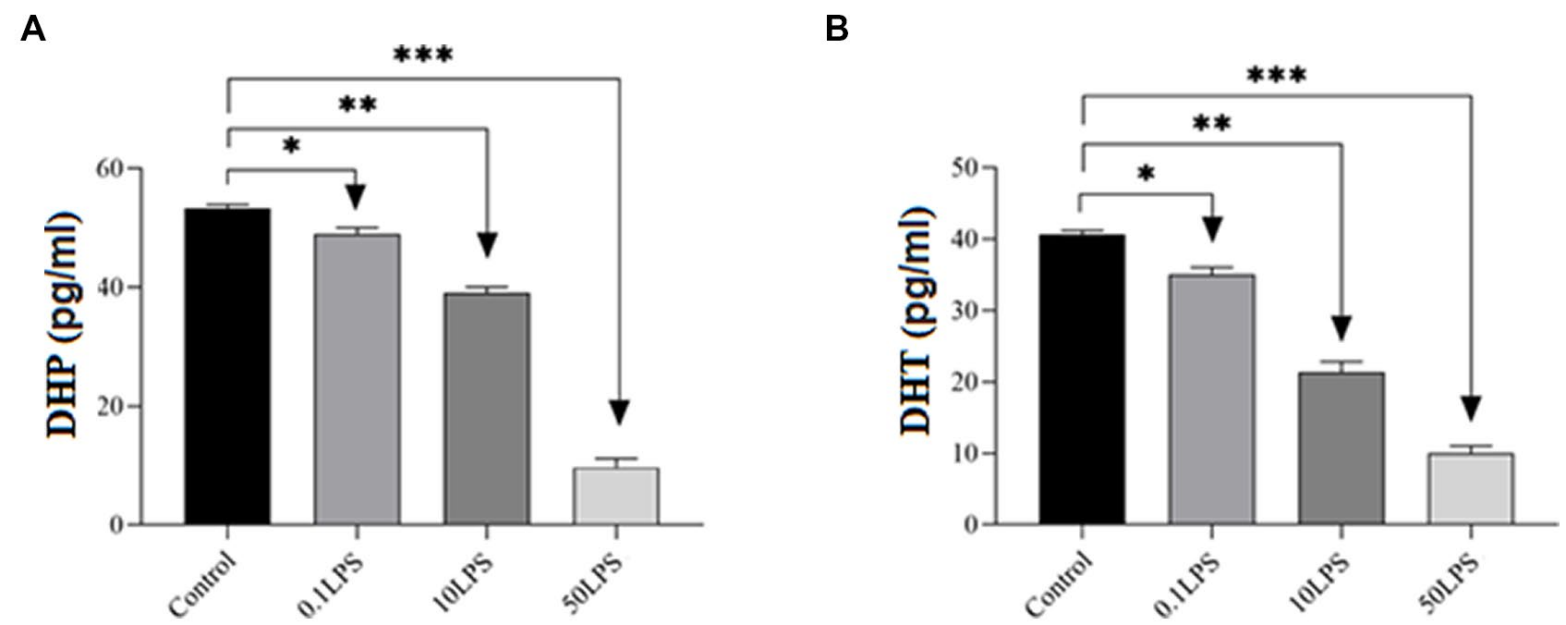
Differences in ELISA results of DHP and DHT levels in SH-SY5Y cells after treatment with different doses of LPS. The ELISA results showed that LPS decreased **(A)** DHP and **(B)** DHT levels in SH-SY5Y cells. Statistical differences between the control and treated groups: *p < 0.05, **p < 0.01, and ***p < 0.001.

LPS decreases DHT and DHP in SH-SY5Y cells. The ELISA results showed that as the LPS concentration increased, the concentrations of DHT and DHP decreased, with the maximum decrease in DHT and DHP concentrations observed at 50 μg/mL of LPS (*p* < 0.001) and a decrease in concentration observed at 0.1 μg/mL of LPS (*p* < 0.05) (Figure 3).

### 3.4. Evaluation of the effect of LPS on apoptosis in SH-SY5Y cell line

**Figure 4:**
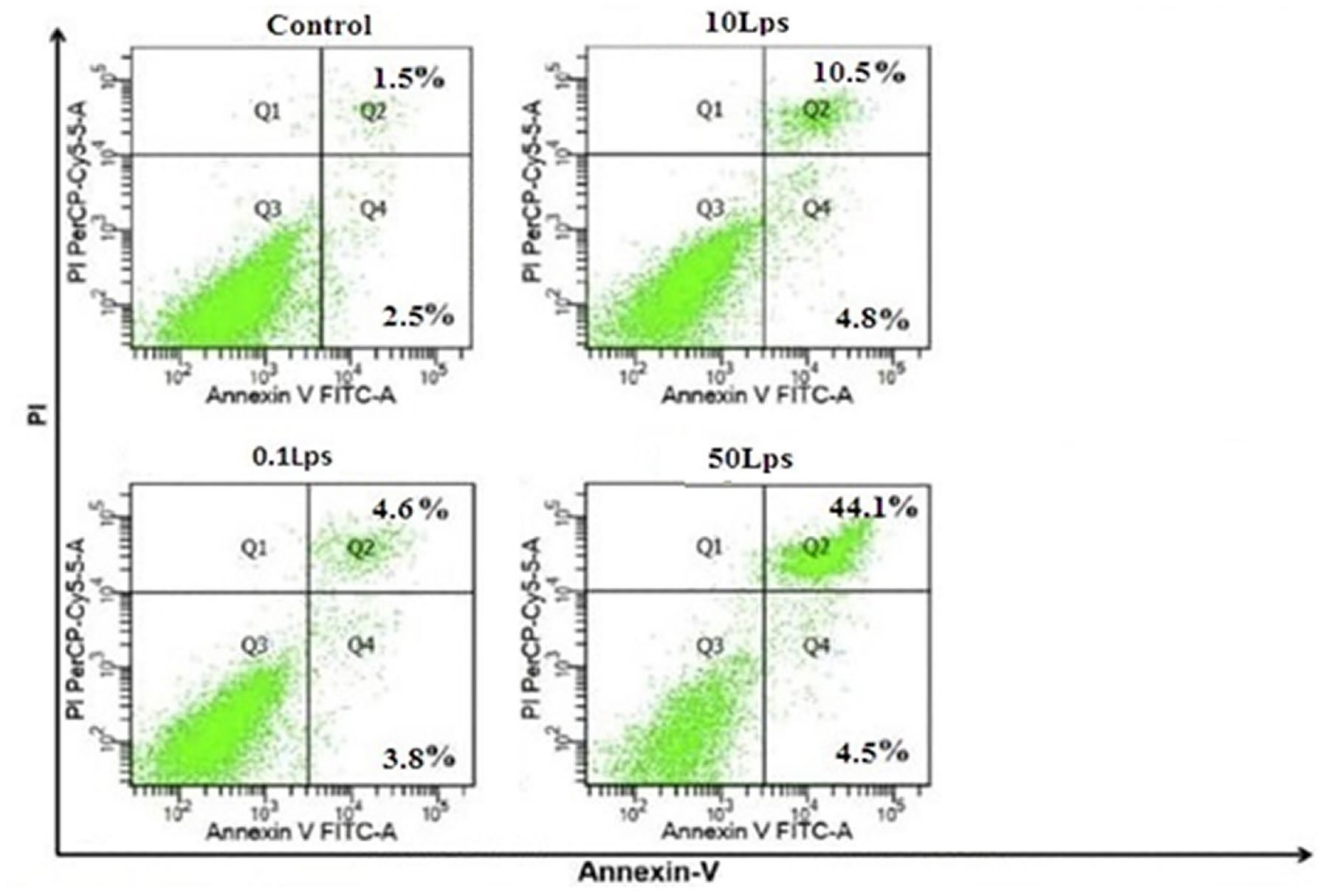
Quantification of apoptosis by Annexin V-FITC/propidium iodide (PI) assay in SH-SY5Y cells treated with LPS dilutions of 0.1, 10, and 50 μg/mL. The induction of apoptosis in SH-SY5Y cells treated with LPS 50 μg/mL was significantly higher than in other treatments. FITC, fluorescein isothiocyanate; PI.

Our study demonstrated that LPS-treated SH-SY5Y cells had significantly higher rates of early and late apoptosis than untreated cells. Furthermore, the total apoptosis rate in SH-SY5Y cells increased with increasing LPS concentration, with rates of 4.6, 10.5, and 44.1% observed for LPS dilutions of 0.1 μg/mL, 10 μg/mL, and 50 μg/mL, respectively. Notably, the overall apoptosis rate in SH-SY5Y cells treated with 50 μg/mL LPS was significantly higher than that of the other treatment groups (Figure 4).

### 3.5. Gene expression changes caused by LPS in SH-SY5Y cell line

**Figure 5:**
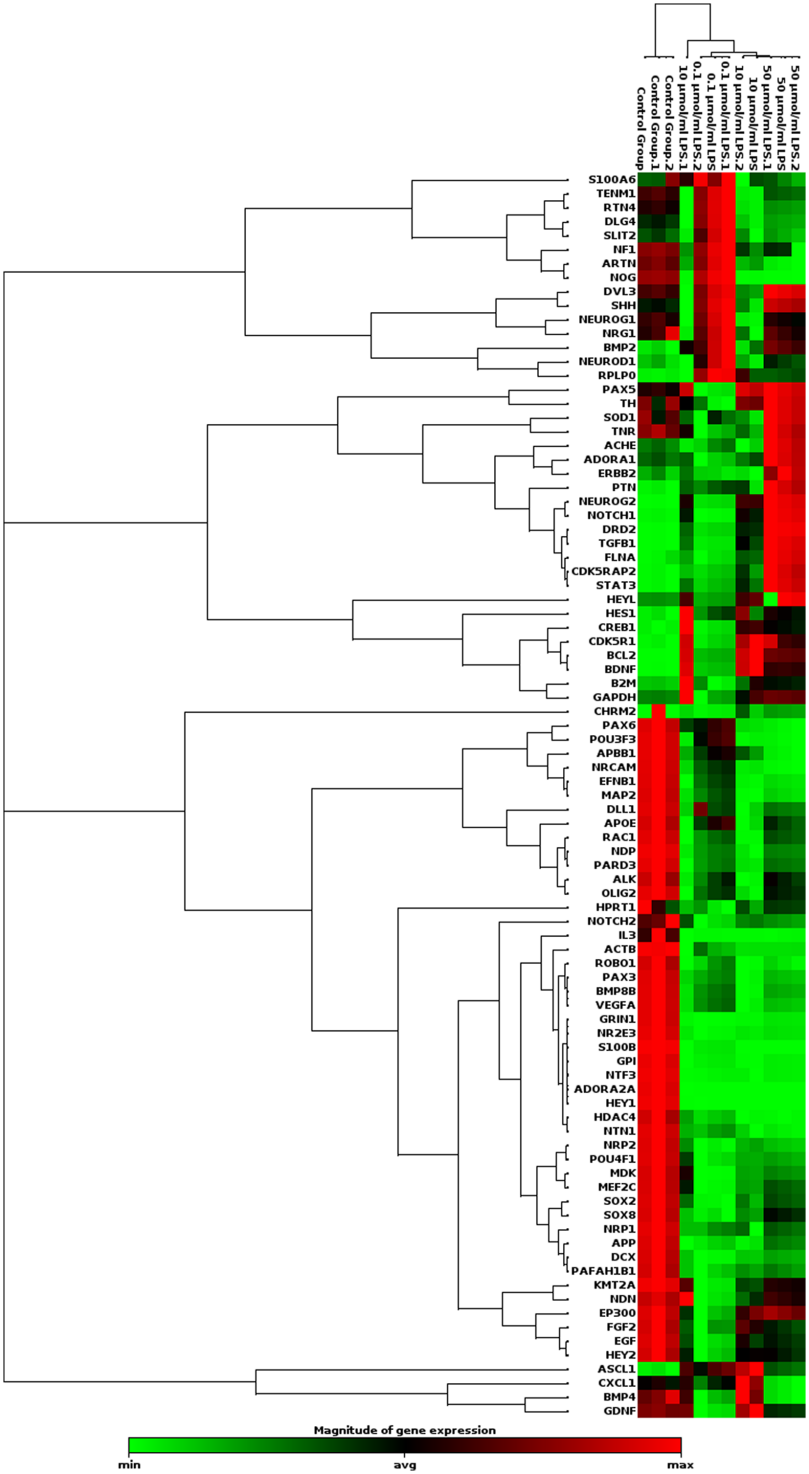
Clustergram analysis of genes involved in human neurogenesis after incubation with different concentrations of LPS in SH-SY5Y cells (*n* = 3). Here, the red color denotes the up regulation of genes across specific samples/conditions, and the green color denotes the downregulation of genes across the specific samples/conditions.

We examined the effect of LPS on gene expression related to neurogenesis. The PCR array analysis indicated that LPS stimulates the expression of genes involved in human neuroblastoma neurogenesis (Table 1). LPS increased the expression of the ASCL1, BCL2, BDNF, NEUROG2, and PTEN genes was increased by 0.1 μg/mL. Additionally, LPS increased the expression of ASCL1, BCL2, BDNF, CDK5R1, CDK5RAP2, CREB1, DRD2, HES1, HEYL, NOTCH1, STAT3, and TGFB1 by LPS 10 μg/mL. Finally, LPS 50 μg/mL upregulated the expression levels of BCL2, BDNF, CDK5R1, CDK5RAP2, CREB1, DRD2, FLNA, NEUROG2, NOTCH1, PTEN, STAT3, and TGFB1 genes (Figure 5).

**Table 1:** PCR array analysis of RT^2^ human neurogenesis genes in ProfilerTM PCR array exposed to different concentrations of LPS compared to the control group.

	0.1LPS		10LPS		50LPS	
Gene	Fold regulation*	p* value	Fold regulation*	*p* value	Fold regulation*	*p* value
ACHE	0.93	0.376	0.78	0.004	1.67	0.001
ADORA1	0.69	0.006	1.01	0.832	1.84	0.001
ASCL1	**2.19**	**0.001**	**2.65**	**0.001**	1.58	0.001
BCL2	**09.17**	**0.001**	**30.63**	**0.001**	**20.75**	**0.001**
BDNF	**4.29**	**0.001**	**27.57**	**0.001**	**17.75**	**0.001**
CDK5R1	1.22	0.071	**4.58**	**0.001**	**3.73**	**0.002**
CDK5RAP2	1.66	0.01	**2.65**	**0.05**	**8.26**	**0.002**
CREB1	1.13	0.206	**4.20**	**0.005**	**3.08**	**0.003**
DRD2	1.67	0.002	**4.84**	**0.004**	**10.98**	**0.005**
FLNA	1.03	0.739	1.75	0.009	**3.98**	**0.007**
HES1	1.52	0.008	**2.09**	**0.046**	1.87	0.002
HEYL	0.85	0.059	**3.10**	**0.004**	0.26	0.244
NEUROG2	**2.22**	**0.001**	09.16	0.003	**20.77**	**0.006**
NOTCH1	0.91	0.279	**7.25**	**0.009**	**13.99**	**0.003**
PTEN	**2.31**	**0.002**	1.94	0.084	**5.12**	**0.002**
STAT3	1.61	0.007	**2.78**	**0.003**	**6.35**	**0.007**
TGFB1	1.93	0.002	**10.00**	**0.004**	**24.70**	**0.006**
RPLP0	1.81	0.001	1.24	0.187	1.28	0.002

*A fold change of more than two was considered acceptable. Statistical significance was established at *p* < 0.05. Fold change of more than two and statistical significance was *p* < 0.05.

## 4. Discussion

Previous studies have shown that LPS causes a decrease in synaptic plasticity, cognitive function, and neuronal function by damaging myelin in (AD) (Cosgrove et al., 2007; Congdon and Sigurdsson, 2018). Furthermore, affecting the inhibitory and excitatory synapses of hippocampal neurons causes loss of function in the CNS (Dai et al., 2021). As a significant ligand, LPS increases the expression of TLR4 and caspase-11 genes by targeting the neuronal TLR4 receptor. Consequently, it causes neuronal cell death in AD by activating the inflammatory response (Duhr et al., 2014; Fu et al., 2019). Because TLR4 expression increases with age and amyloid beta levels, the interaction between LPS and TLR4 could influence the development of AD (Fulop et al., 2018). Although the general mechanism of AD is not fully understood, the role of amyloid beta toxicity in the pathogenesis of AD is vital (Garcia-Ovejero et al., 2005). Our study showed that different concentrations of LPS can have cytotoxic effects on SH-SY5Y cells, and increased amyloid beta deposition can exacerbate apoptosis in SH-SY5Y cells.

Cholesterol is essential as a basis for producing steroid hormones, including progesterone and estrogen, and these two hormones play various physiological functions in both men and women. Because of the critical role of progesterone and estrogen in neurodegenerative diseases, including AD, many studies have investigated changes in these two hormones. Some studies have shown that the use of estrogen reduces the risk of AD in women. Therefore, estrogen compounds may be used to treat AD in the elderly (Gascón et al., 2016; Grothe et al., 2017). However, because hormone therapy is expensive and lengthy, patients often abandon treatment (He et al., 2014). Some studies have shown that testosterone and progesterone play an influential role in neuroprotection during the early stages of AD development. However, it is challenging to apply these two hormones effectively in the later phases of the disease (Heinrich et al., 2014; Huo et al., 2016; Hung et al., 2017; He et al., 2019). Furthermore, DHT has been found to modulate the expression of Aβ, the caspase-3, Bcl-2 and Bax, and synaptophysin, as well as reduce neuronal damage in mice treated with LPS. DHT also exerts anti-neuroinflammatory and neuroprotective effects, making androgen replacement therapy a potential therapeutic strategy for improving cognitive and behavioral function in neuroinflammation-related diseases (Yang et al., 2020). Our study revealed that an increase in LPS concentration led to a decrease in both DHT and DHP concentrations. The maximum decrease in DHT and DHP concentration occurred at an LPS concentration of 50 μg/mL. Previous research has shown that LPS in the hypothalamus or pituitary gland can disrupt follicular growth and function in mice by suppressing gonadotropin release (Kuo et al., 2010; Kitagishi and Matsuda, 2013; Johnson et al., 2016). Additionally, in follicles with high LPS compared to those with low LPS, there was a suppression of CYP17 gene expression in theca cells and P450 aromatase gene expression in granulosa cells, resulting in decreased estrogen levels (E2) (Lin et al., 2010; Lee et al., 2014; Liu et al., 2017). LPS has also been shown to reduce progesterone biosynthesis in mice (Mao and Sun, 2015; Masters et al., 2015). These findings suggest that menopause and the decrease in sex hormones, such as estrogen and progesterone in women and testosterone in men, may increase the susceptibility to AD in old age.

0.1 μg/mL LPS increased the expression of ASCL1, BCL2, BDNF, NEUROG2, and PTEN. LPS (10 μg/mL) increased the expression of ASCL1, BCL2, BDNF, CDK5R1, CDK5RAP2, CREB1, DRD2, HES1, HEYL, NOTCH1, STAT3, and TGFB1. 50 μg/mL LPS increased the expression levels of BCL2, BDNF, CDK5R1, CDK5RAP2, CREB1, DRD2, FLNA, NEUROG2, NOTCH1, PTEN, STAT3, and TGFB1. The results of our study show that LPS concentration has a differential effect on the expression of genes related to neurogenesis in human neuroblastoma. Increasing LPS concentration can significantly affect the expression of neurogenesis-related genes in human neuroblastoma. In AD, neuron destruction is triggered by increased CDK5 activity. CDK5 kinase, abundantly expressed in neurons and plays a critical role in synaptic plasticity and neuronal development, is implicated in triggering neuronal destruction in AD through increased activity (Pan et al., 2016; Park et al., 2021). While CDK5 overactivity is linked to the development of neurodegeneration, it also plays a crucial role in various physiological functions, including migration, neuroblasts, and synaptic plasticity. CDK5 is located at the end of the axon growth cone, where it regulates the growth of neural progenitor cells into mature neurons, making it necessary for the maturation phase of neurogenesis. Altered CDK5 activity in neural progenitor cells is associated with defects in neurogenesis in AD (Pascual et al., 2015). We also observed increased expression of genes, including FLNA and STAT3, both of which function as cofactors and transcription factors. Therefore, FLNA is considered associated with the pathogenesis of amyloid beta and tau proteins in AD (Pitsavos et al., 2006; Patricia et al., 2013). Amyloid beta induces the production of this protein, which plays a crucial role in the AD signaling pathway. Persistent activation of the TLR4 receptor by beta-amyloid causes an overproduction of inflammatory cytokines and triggers neuroinflammation (Pompili et al., 2012; Ruigrok et al., 2014; Robinson et al., 2016; Schöll et al., 2016). Furthermore, it is understandable that the limitations of cell lines in mimicking AD and the events that occur in AD make the field stronger for detailed studies in animal models (Zhan et al., 2018).

## 5. Conclusion

Different concentrations of LPS can have cytotoxic effects on SH-SY5Y cells, and increasing amyloid beta deposition may enhance apoptosis in these cells. Moreover, LPS can reduce the concentration of DHT and DHP. Changes in the expression of genes related to neurogenesis in human neuroblastoma cells under the influence of LPS suggest novel sub-mechanisms. Targeting DHT and DHP or neurogenesis in human neuroblastoma cells may be a promising therapeutic strategy for AD treatment or symptom relief. However, further studies are needed to fully explain the underlying mechanism.

## Data availability statement

The raw data supporting the conclusions of this article will be made available by the authors, without undue reservation.

## Ethics statement

The Ethics Committee of the Tabriz University of Medical Sciences approved the study protocols.

## Author contributions

AN and FK-K were involved in the study design. SN and BS contributed to the analysis and interpretation of data. BS, RR, and MN contributed to revising the manuscript content. AN gave consent for the final version. SR wrote the manuscript. All authors contributed to the article and approved the submitted version.

## Funding

This work was supported by a grant from the Vice Chancellor for Research, Tabriz University of Medical Sciences, Tabriz, Iran, with the grant number IR.TBZMED.REC.1396.896.

## Conflict of interest

The authors declare that the research was conducted in the absence of any commercial or financial relationships that could be construed as a potential conflict of interest.

## Publisher’s note

All claims expressed in this article are solely those of the authors and do not necessarily represent those of their affiliated organizations, or those of the publisher, the editors and the reviewers. Any product that may be evaluated in this article, or claim that may be made by its manufacturer, is not guaranteed or endorsed by the publisher.
